# Evidence of Active Pro-Fibrotic Response in Blood of Patients with Cirrhosis

**DOI:** 10.1371/journal.pone.0137128

**Published:** 2015-08-28

**Authors:** Gloria Sanchez-Antolín, Carolina Almohalla-Alvarez, Pilar Bueno, Raquel Almansa, Verónica Iglesias, Lucia Rico, Alicia Ortega, Eva Muñoz-Conejero, Felix García-Pajares, Jesus F. Bermejo-Martin

**Affiliations:** 1 Unidad de Hepatología, Unidad de Trasplante Hepático. Hospital Universitario Río Hortega, Valladolid, Spain; 2 Servicio de Análisis Clínicos, Hospital Clínico Universitario de Valladolid, Valladolid, Spain; 3 Investigación Médica en Infección e Inmunidad (IMI), Hospital Clínico Universitario de Valladolid-IECSCYL, Valladolid, Spain; 4 Escuela de Enfermería Universidad de Valladolid, Valladolid, Spain; University of Navarra School of Medicine and Center for Applied Medical Research (CIMA), SPAIN

## Abstract

The role of systemic immunity in the pathogenesis of cirrhosis is not fully understood. Analysis of transcriptomic profiles in blood is an easy approach to obtain a wide picture of immune response at the systemic level. We studied gene expression profiles in blood from thirty cirrhotic patients and compared them against those of eight healthy volunteers. Most of our patients were male [n = 21, 70%] in their middle ages [57.4 ± 6.8 yr]. Alcohol abuse was the most frequent cause of cirrhosis (n = 22, 73%). Eleven patients had hepatocellular carcinoma (36.7%). Eight patients suffered from hepatitis C virus infection (26.7%). We found a signature constituted by 3402 genes which were differentially expressed in patients compared to controls (2802 over-expressed and 600 under-expressed). Evaluation of this signature evidenced the existence of an active pro-fibrotic transcriptomic program in the cirrhotic patients, involving the [extra-cellular matrix (ECM)-receptor interaction] & [TGF-beta signaling] pathways along with the [Cell adhesion molecules] pathway. This program coexists with alterations in pathways participating in [Glycine, serine and threonine metabolism], [Phenylalanine metabolism], [Tyrosine metabolism], [ABC transporters], [Purine metabolism], [Arachidonic acid metabolism]. In consequence, our results evidence the co-existence in blood of a genomic program mediating pro-fibrotic mechanisms and metabolic alterations in advanced cirrhosis. Monitoring expression levels of the genes involved in these programs could be of interest for predicting / monitoring cirrhosis evolution. These genes could constitute therapeutic targets in this disease.

## Introduction

Intra-hepatic inflammation and immune response are known to play central roles in the pathogenesis of cirrhosis [1]. Both are direct causes of injury to hepatocytes and activation of hepatic stellate cells (HSCs), which ultimately leads to liver fibrosis[2]. In contrast, the participation of the systemic immunological response in the pathogenesis of this disease has not been totally clarified. Analysis of transcriptomic profiles in peripheral blood is a widely accepted method to evaluate the complex immune networks that operate throughout the entire body. It has proven to be a valid approach for studying pathogenesis as well as to identifying potential biomarkers in a large variety of diseases[3]. While there are a number of previous works evaluating gene expression in hepatic tissue of cirrhotic patientsor chronic liver diseases [4][5][6][7], there is very limited information on the transcriptomic profiles present in blood of these patients.

Our work unveils for the first time the presence of an active pro-fibrotic transcriptomic program in the blood of patients with liver cirrhosis, co-existing with alterations in the metabolome. Our findings evidence the important impact of cirrhosis on the immune response at the systemic level, providing also interesting clues on the potential roles of peripheral leukocytes in the pathogenesis of this disease.

## Materials and Methods

### Patients

We studied thirty cirrhotic patients of the Hepatology Unit of Hospital Univesitario Rio Hortega, Valladolid, Spain. We compared the study group with eight healthy volunteers who were in the same age range recruited from the staff of the Hospital, as control group. Approval of the study protocol for both the scientific and ethical aspects was obtained from the Scientific Committee for Clinical Research of “Hospital Universitario Río Hortega” (Comité de Ética en Investigación Clínica, CEIC-Área Oeste, Valladolid, Spain), conforming to the ethical guidelines of the 1975 Declaration of Helsinki. Written informed consent was obtained from all participants before recruitment.

### Sample collection

A sample of 2.5 mL of blood was collected from each patient using PaxGene venous blood vacuum collection tubes (Becton Dickinson, USA).

### Microarray processing and data analysis

RNA extraction and processing for microarray analysis was performed as previously described by our group [8]. Data analysis was done using GeneSpring GX 12.0 software. The original data were cleaned and normalised in three steps: 1) local background was subtracted from the individual spot intensity; 2) log-transformed signal intensity values were globally normalised using the percentile shift algorithm, shifting to the 75th percentile of each sample, for per chip normalisation; and 3) baseline transformation of the data was performed using the median of all samples. Before statistical analyses, all microarrays were subjected to quality and filtering criteria. The quality of the microarray data was assessed on principal component analysis plots. Student’s t-tests (GeneSpring GX12.0) were used to identify genes that were differentially expressed between groups at a level of significance *p*< 0.05, with Benjamini-Hochberg multiple testing corrections and fold changes ≥ 2. DAVID (Database for Annotation, Visualization, and Integrated Discovery) & KEGG (Kyoto Encyclopedia of Genes and Genomes) were used to select, annotate and visualize genes by function and pathway[9][10]. Graphite Web software was used to represent signalling pathways present in the analysis[11]. The resulting microarray data sets were uploaded at the Array Express microarray data repository (E-MTAB-3338). Gene expression results from microarrays were validated using the Bio-Rad QX200 Droplet Digital PCR system as previously described[12]. Primers and probes were purchased to Life Technologies (Custom TaqMan assays with probes for problem genes labeled in FAM and housekeeping gene (GAPDH) labeled in VIC) (S1 Fig).

## Results

### Patients`clinical characteristics (Table 1)

**Table 1:** Clinical characteristics of the patients.

	TOTAL COHORT (n = 30)	HCC (n = 11)	NO HCC (n = 19)	*p*	NRV
**Age (mean ± SD)**	57.4 ± 6.8	58.3 ± 9.4	56.8 ±7.1	n.s.	n.a
**Male, n(%)**	21 (70)	8 (27)	13 (43)	n.s.	n.a
**HCV infection**	3 (10)	1 (3.3)	2 (6.4)	n.s.	n.a
**Alcoholism**	17(58)	6 (20)	11 (38)	n.s.	n.a
**HCV+Alcoholism**	5 (16)	3 (10)	2 (6)	n.s.	n.a
**Other causes**	5 (16)	1 (3.3)	4 (12.8)	n.s.	n.a
**Diabetes Mellitus II**	8 (26)	3 (10)	5(16)	n.s.	n.a
**High blood presure**	11 (36)	6(20)	5 (16)	n.s.	n.a
**MELD score**	11.8±3.8	9.5 ± 2.7	13.2 ±3.7	**0.015**	n.a
**Hemoglobin (g/dl)**	11.5±2.4	12.6 ± 2.7	10.8 ±1.9	n.s.	13.2–16.8
**Leukocytes (x10** ^**3**^ **/μl)**	5243.3±3328	4572.7 ± 2038	5631.6 ±3887.2	n.s.	4–10.5
**Neutrophil (%)**	64.2±16.4	63.3 ± 17.8	64.6 ±15.8	n.s.	41–72
**Platelet (x10** ^**3**^ **/μl)**	91,5±48,9	89,2± 35,0	94,6 ±56,1	n.s.	150–350
**ProthrombinTime (seconds)**	78.6±20.2	88.6 ± 16.3	72.7 ± 20.4	**0.030**	60–140
**INR**	1.2±0.3	1.1 ± 0.2	1.3 ± 0.4	**0.007**	0.8–1.2
**Glucose (mg/dl)**	142.6±76.7	155 ± 87.0	134.8 ± 68.9	n.s.	82–115
**Albumin (g/dl)**	3.4±0.8	3.5 ± 0.6	2.9 ± 0.8	**0.006**	3.5–4.6
**Bilirrubin (mg/dl)**	2.5±1.8	2.0 ± 1.3	2.9 ± 2.0	**0.021**	0.2–1.1
**Cholesterol (mg/dl)**	150.5±43.5	159.4 ± 32.9	143.6 ± 54.5	n.s.	<200
**Triglycerides (mg/dl)**	96.5±42.4	100.8± 51.3	83.1 ± 30.6	n.s.	<150
**C Reactive Protein**	30.8±81.6	12.9 ± 25.7	50.0 ± 117.8	n.s.	0–10
**Uric Acid (mg/dl)**	6.9±2.6	6.3± 2.2	6.7 ± 3.2	n.s.	2.6–6
**AST (U/L)**	78.0±92.7	73 ± 45.4	84.0 ± 108.2	n.s.	0–50
**ALT (U/L)**	66±55.6	87 ± 75.9	58.0± 34.9	n.s.	1.0–55
**GGT (U/L)**	160.1±203.9	173.8 ± 224.5	136.4 ± 185.5	n.s.	0–55
**ALP (U/L)**	190.4±171.5	169.4 ± 91.3	209.5 ± 224.6	n.s.	30–120

HCV: Hepatitis C Virus;HCC: hepatocellular carcinoma; AST: aspartate aminotransferase; ALT: alanine aminotransferase; GGT: gamma glutamyl transpeptidase; ALP: alkaline phosphatase; INR: International Normalized Ratio coagulation index; MELD (Model for End-stage Liver Disease) score; NRV: normal reference values. N.a: not applicable.

Most of our patients were male in their middle ages. Alcohol abuse was the most frequent cause of cirrhosis in our cohort. There were eleven patients with hepatocellular carcinoma (HCC) (36.6%). Eight patients suffered from HCV infection (26.6%). The most frequent co-morbidities were diabetes mellitus and high blood pressure. The mean MELD (Model for End-Stage Liver Disease) score was 11.8, being the highest in the group of patients with no HCC. Patients with no HCC had lower albumin values and higher bilirrubin levels. These patients showed in addition increased INR values. As expected, both groups of patients showed elevated levels of liver enzymes and also of C-reactive protein in serum. In turn, both had platelet counts in blood below normality.

### Gene expression analysis

**Fig 1:**
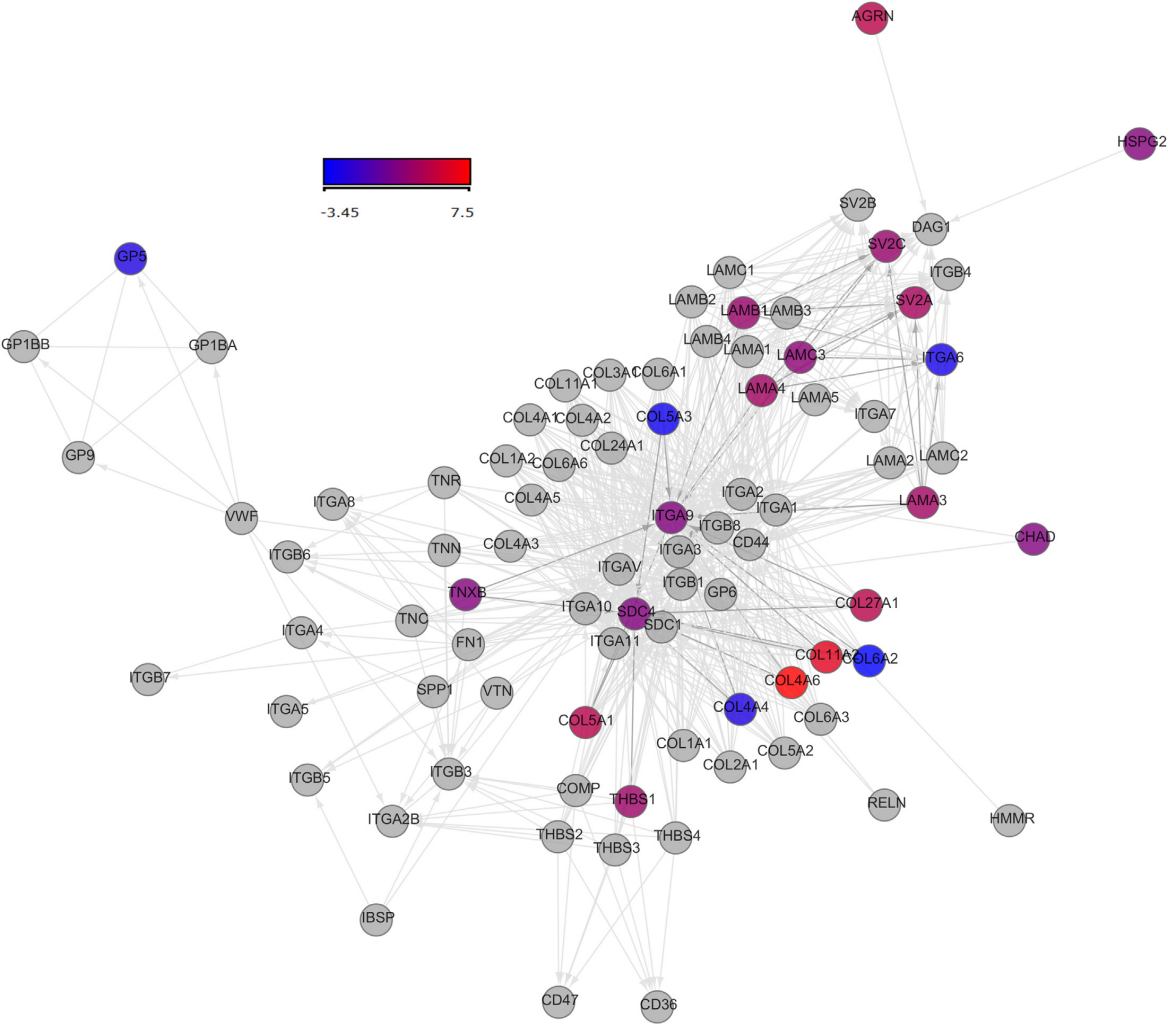
Network analysis representing genes participating in the [extra-cellular matrix-receptor interaction] pathway. Coloured nodes represent the portions of the pathways mostly involved in cirrhosis. The colour of the nodes is proportional to their fold change(FC) compared to healthy controls, with the scale ranging from -3.4 FC (blue) to 7.5 FC (red).

**Fig 2:**
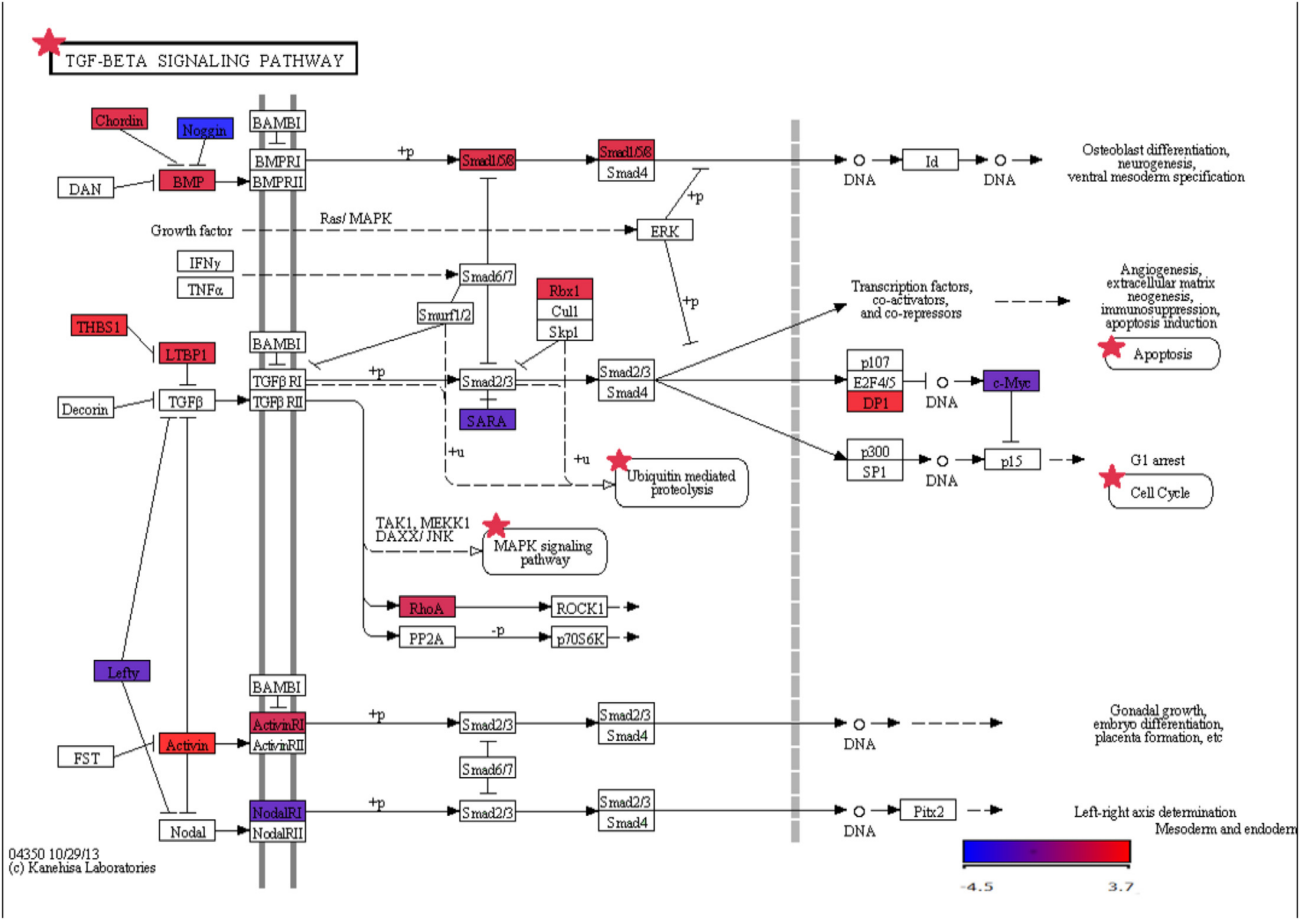
Kyoto Encyclopedia of Genes and Genomes diagram representing the [Transforming Growth Factor-beta] signaling pathway. Those genes differentially expressed between patients and controls are showed by a color scale.

As said above, MELD score was lower in patients with HCCcompared with that of patientswith no HCC. Since this could influence gene expression profiles (GEP), we compared GEP in both groups of patients. Analysis revealed no differences between both groups regarding gene expression levels, and both groups were thus considered as a single one. Next, we compared the group of patients against healthy controls. This comparison revealed 3402 genes differentially expressed between patients with cirrhosis and controls (2802 up-regulated and 600 down regulated) (S1 Table). DAVID/KEGG analysis revealed that these genes participate in three major groups of intracellular signaling pathways (S2 Table)(we highlight below those pathways identified by the analysis at the level *p*< 0.05):

- **Signaling pathways involved in fibrogenesis**: [extra-cellular matrix (ECM)-receptor interaction] (Fig 1), [Transforming Growth Factor beta (TGF-beta) signaling pathway] (Fig 2).
- **Signaling pathways involved in cell adhesion:** [Cell adhesion molecules (CAMs)]
- **Signaling pathways involved in metabolism:** [Glycine, serine and threonine metabolism], [Phenylalanine metabolism], [Tyrosine metabolism], [ABC transporters], [Purine metabolism], [Arachidonic acid metabolism]

## Discussion

Our study demonstrates that cirrhosis has an important impact on blood, evidencing the existence of an active transcriptomic program in circulating leukocytes, characterized by an enriched response in transcripts associated to fibrogenesis. This way, patients had a marked activation of the [ECM-receptor interaction)] pathway. The extracellular matrix consists of a complex mixture of structural and functional macromolecules and serves an important role in tissue and organ morphogenesis and in the maintenance of cell and tissue structure and function. Patients showed active production of three heparan-sulfate proteins pertaining to this pathway: Agrin (AGRN),heparan sulfate proteoglycan 2(HSPG2) and syndecan 4 (SDC4). Heparan sulfate chains, the highly sulfated glycosaminoglycans attached to the protein core of heparan sulfate proteoglycans, are intricately involved in a variety of physiological processes such as morphogenesis, wound healing, regulation of cell differentiation and growth, and establishment of cell–cell and cell–extracellular matrix contacts [13]. They accumulate in the matrix of the expanding scar tissue in chronic liver diseases [14]. In fact, agrin accumulates in the liver of patients with cirrhosis, and has been proposed to play a stimulatory role in neoangiogenic processes, and a supportive role in bile ductule proliferation [14]. SDC4 has been proposed to be a sensor of tension exerted on the extracellular matrix, which is an important event in wound contraction, tumor-stroma interactions, fibrosis and the regulation of motility[15]. In mice, SDC4 knockout protects against tubulointerstitial fibrosis in the kidney [16]. Expression of thrombospondin 1 (THBS1) and of three laminin genes (LAMC3, LAMA3/ LAMA4, LAMB1) was activated also in our patients. It has been described that level of thrombospondin-1 (THBS-1) is higher in the serum of patients with hepatic fibrosis [17]. In turn, laminin is a major serum marker of liver fibrosis[18]. Patients had also increased expression of tenascin XB (TNXB). Tenascins constitute a family of matricellular proteins, primarily modulating interactions of cells with other matrix components and growth factors. Tenascin X has been shown to affect TGF-β activation and signalling, suggesting that these proteins might be important factors in fibrosis development[19]. Serum levels of Tenascin have been reported to reflect hepatic fibrogenesis and inflammation [20]. Patients with cirrhosis showed increased expression in blood of eight genes coding for collagen synthesis COL4A6, COL5A1, COL11A2, COL12A1, COL27A1, COL28A1, COL23A1, COL14A1. Although three other genes (COL4A4, COL6A2, COL5A3) were down-regulated in these patients, our results overall support the notion that white blood cells from patients with cirrhosis are predominately prone to produce collagen. Collagen genes are involved in activation of Hepatic Stellate Cells (HSCs), along with a number of genes also found to be up-regulated in our cohort of patients: colony stimulating factor 1 (macrophage) (CSF1), endothelin 1 (EDN1), fibroblast growth factor 1 (FGF1), insulin-like growth factor 2 (IGF2), lymphotoxin alpha (LTA), lymphocyte antigen 96 (LY96), myosin, heavy chain 14 (MYH14), myosin, light chain 4 (MYL4). In the liver, activation of HSCs leads to collagen deposition and hepatic fibrogenesis [2][21]. IGF2 (somatomedin A) induces in addition proliferation of hepatocytes [22].

TGF-beta signaling pathway was also activated in the group of patients, with up-regulation of growth differentiation factor 7 (GDF7), inhibin, beta C(INHBC), ras homolog family member A(RHOA), latent transforming growth factor beta binding protein 1 (LTBP1), SMAD family member 5 (SMAD5), SMAD family member 9 (SMAD9), retinoblastoma-like 1 (p107) (RBL1), transcription factor Dp-1(TFDP1), THBS1, growth differentiation factor 5 (GDF5), inhibin, beta E(INHBE),chordin(CHRD) andring-box 1, E3 ubiquitin protein ligase(RBX1). Activation of this pathway is one of the most important stimuli for extracellular matrix synthesis at the systemic level, and also in the liver [23].

The top signaling pathway identified by KEGG was [Cell Adhesion molecules]. Genes of this pathway were up-regulated in their vast majority, supporting that leukocytes from cirrhotic patients are not only activated to mediate fibrogenesis, but also to leave circulation and interact with the extracelular matrix components[24].

We found also increased expression of caveolin-1 and caveolin-2 in blood in the group of patients with cirrhosis. Yokomori *et al* have recently reported caveolin-1 in liver sinusoidal endothelial cells to correlate with cirrhosis progression [25]. Caveolin-1 (CAV-1) is involved in hepatic sinusoidal angiogenesis and remodeling during progression to cirrhosis[25].

Severity of liver fibrosis in known to be associated to alterations in the metabolome[26][27][28]. According to this, our work demonstrates that cirrhosis induces a wide re-programming of transcriptomic signatures involved in metabolism, as evidenced by the observed alterations in the pathways participating in aminoacid, nucleotide and arachidonic acid metabolism.

Although there are limited works on gene expression profiles in blood from patients with liver diseases, some information is available. Shi M *et al*found a three-gene expression signature associated to early human hepatocellular carcinoma[29], constituted by the chemokine (C-X-C motif) receptor 2 (CXCR2), C–C chemokine receptor type 2 (CCR2) and the E1A-Binding Protein P400 (EP400). We failed to find this signature in our cohort of patients, neither comparing the total cohort with the controls, not when this comparison was performed considering just those patients with HCC and controls. Differences in the nature of the two works could explain this. The study of Shi *et al* was performed using RNA from peripheral blood mononuclear cells (PBMCs), while our study analyzed RNA from whole blood. In consequence, gene transcripts from neutrophils were considered in our work, but not in that from Shi *et al*.

In conclusion, our results evidence that cirrhosis is characterized by the presence of an active pro-fibrotic program at the transcriptomic level in blood. This program could be contributing to the pathogenesis of this disease and also to that of its extra-hepatic complications (i.e atherosclerosis, cardiovascular and brain disease). Blood is a sample much easier to access than liver biopsies. Monitoring the expression levels in blood of the genes participating in thesignatures identified in this work could be of interest for predicting / monitoring cirrhosis evolution and their associated metabolic complications. In addition, further works should elucidate if the genes identified here could constitute therapeutic targets in this disease.

## Supporting Information

S1 Fig PCR validation of microarray data. Expression values obtained from the microarrays for COL27A1 and COL5A1 showed a significant positive correlation, confirmed by using digital PCR. FC: Fold Change.
(TIF)

S1 Table List of genes differentially expressed genes between patients and controls. FC: fold change.
(XLSX)

S2 Table Database for Annotation, Visualization, and Integrated Discovery & Kyoto Encyclopedia of Genes and Genomes analysis showing the main intracellular signaling pathways differentially expressed between patients and controls. (DOCX)
