# Supplementary figures and images for: Evidence of Active Pro-Fibrotic Response in Blood of Patients with Cirrhosis
Source: PLoS One. 2015 Aug 28;10(8):e0137128. doi: 10.1371/journal.pone.0137128 (PMC4552880; doi:10.1371/journal.pone.0137128)

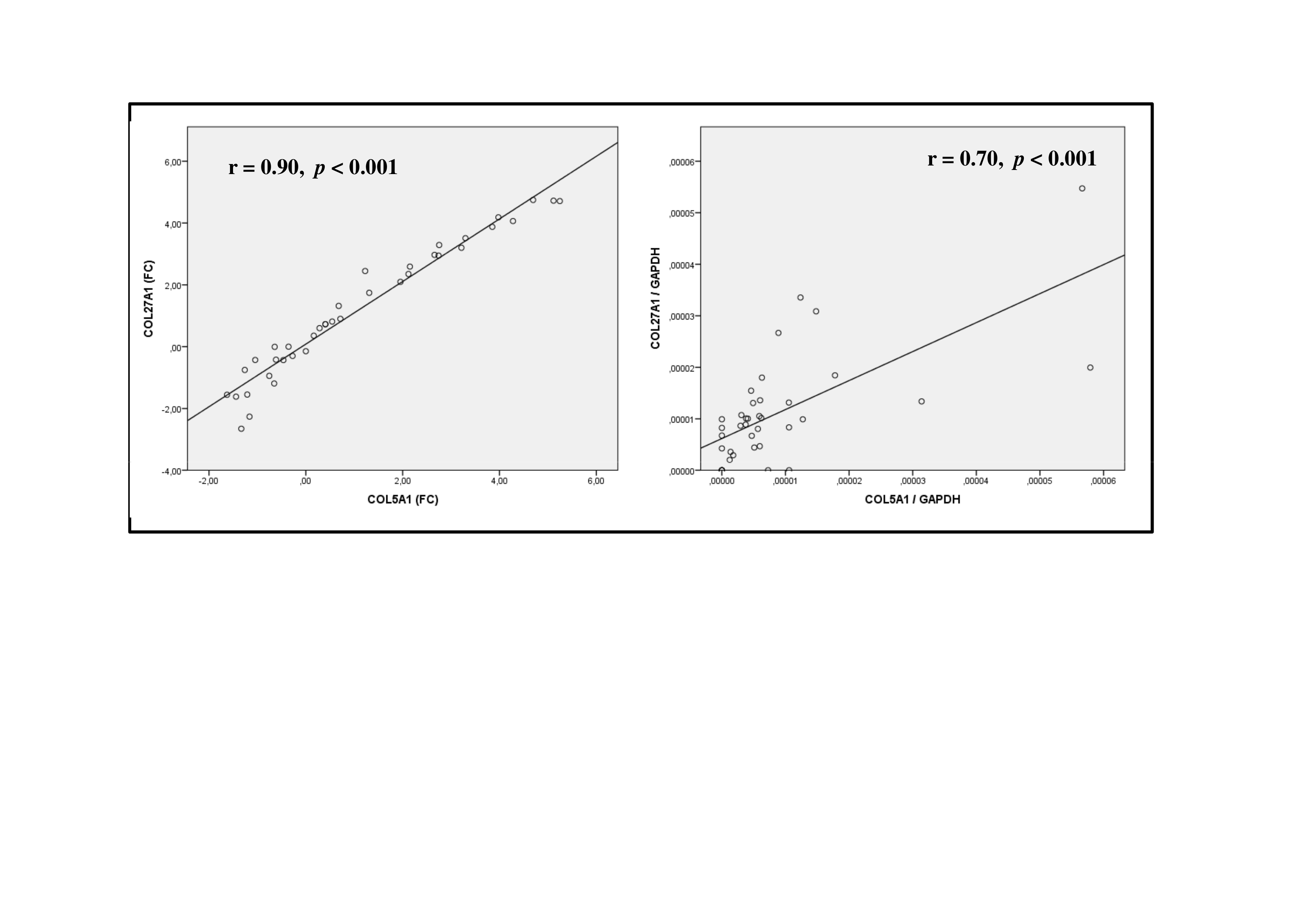

Supplement: S1 Fig — Expression values obtained from the microarrays for COL27A1 and COL5A1 showed a significant positive correlation, confirmed by using digital PCR. FC: Fold Change. (TIF) [file pone.0137128.s001.tif]
