# Supplementary material for: Evidence of Active Pro-Fibrotic Response in Blood of Patients with Cirrhosis
Source: PLoS One. 2015 Aug 28;10(8):e0137128. doi: 10.1371/journal.pone.0137128 (PMC4552880; doi:10.1371/journal.pone.0137128)
Supplement: S2 Table — (DOCX) [file pone.0137128.s003.docx]

| **KEGG PATHWAY** | **KEGG**  ***P value*** | **Agilent probe code** | **Fold Change** | **Gene Symbol** | **Description** |
| --- | --- | --- | --- | --- | --- |
| Cell adhesion molecules (CAMs) | 0.003 | A_23_P109034 | 2.056 | SDC4 | Homo sapiens syndecan 4 (SDC4), mRNA [NM_002999] |
|  |  | A_23_P115161 | 6.166 | DARC | Homo sapiens Duffy blood group, chemokine receptor (DARC), transcript variant 2, mRNA [NM_002036] |
|  |  | A_23_P159335 | -2.899 | CD8B | Homo sapiens CD8b molecule (CD8B), transcript variant 5, mRNA [NM_004931] |
|  |  | A_23_P252193 | 2.086 | ITGA9 | Homo sapiens integrin, alpha 9 (ITGA9), mRNA [NM_002207] |
|  |  | A_23_P311875 | -2.394 | CD6 | Homo sapiens CD6 molecule (CD6), mRNA [NM_006725] |
|  |  | A_23_P317667 | 2.628 | ICOSLG | Homo sapiens inducible T-cell co-stimulator ligand (ICOSLG), mRNA [NM_015259] |
|  |  | A_23_P411113 | 3.032 | CNTNAP1 | Homo sapiens contactin associated protein 1 (CNTNAP1), mRNA [NM_003632] |
|  |  | A_23_P47410 | 2.280 | ESAM | Homo sapiens endothelial cell adhesion molecule (ESAM), mRNA [NM_138961] |
|  |  | A_23_P49155 | 3.009 | CDH3 | Homo sapiens cadherin 3, type 1, P-cadherin (placental) (CDH3), mRNA [NM_001793] |
|  |  | A_23_P76034 | 2.721 | PVRL1 | Homo sapiens poliovirus receptor-related 1 (herpesvirus entry mediator C) (PVRL1), transcript variant 3, mRNA [NM_203286] |
|  |  | A_24_P252364 | -2.827 | NRCAM | Homo sapiens neuronal cell adhesion molecule (NRCAM), transcript variant 1, mRNA [NM_001037132] |
|  |  | A_24_P295999 | 2.708 | CD4 | Homo sapiens CD4 molecule (CD4), transcript variant 1, mRNA [NM_000616] |
|  |  | A_24_P320033 | 2.950 | CD80 | Homo sapiens CD80 molecule (CD80), mRNA [NM_005191] |
|  |  | A_24_P385313 | 2.583 | PTPRF | Homo sapiens protein tyrosine phosphatase, receptor type, F (PTPRF), transcript variant 1, mRNA [NM_002840] |
|  |  | A_24_P86993 | -2.054 | JAM3 | Homo sapiens junctional adhesion molecule 3 (JAM3), transcript variant 1, mRNA [NM_032801] |
|  |  | A_32_P163247 | -2.656 | CD8A | Homo sapiens CD8a molecule (CD8A), transcript variant 1, mRNA [NM_001768] |
|  |  | A_33_P3217393 | 2.489 | CD276 | Homo sapiens CD276 molecule (CD276), transcript variant 1, mRNA [NM_001024736] |
|  |  | A_33_P3231447 | -2.528 | ITGA6 | Homo sapiens integrin, alpha 6 (ITGA6), transcript variant 2, mRNA [NM_000210] |
|  |  | A_33_P3237574 | 2.205 | HLA-A | Homo sapiens major histocompatibility complex, class I, A (HLA-A), transcript variant 2, mRNA [NM_001242758] |
|  |  | A_33_P3239884 | 2.623 | NCAM2 | Homo sapiens neural cell adhesion molecule 2 (NCAM2), mRNA [NM_004540] |
|  |  | A_33_P3250680 | -2.369 | CD40LG | Homo sapiens CD40 ligand (CD40LG), mRNA [NM_000074] |
|  |  | A_33_P3272823 | 2.039 | MAG | Homo sapiens myelin associated glycoprotein (MAG), transcript variant 1, mRNA [NM_002361] |
|  |  | A_33_P3285540 | -2.495 | CLDN5 | Homo sapiens claudin 5 (CLDN5), transcript variant 1, mRNA [NM_001130861] |
|  |  | A_33_P3299739 | 2.775 | CLDN19 | Homo sapiens claudin 19 (CLDN19), transcript variant 2, mRNA [NM_001123395] |
|  |  | A_33_P3314401 | 4.001 | CLDN16 | Homo sapiens claudin 16 (CLDN16), mRNA [NM_006580] |
|  |  | A_33_P3341970 | 2.913 | NEGR1 | Homo sapiens neuronal growth regulator 1 (NEGR1), mRNA [NM_173808] |
|  |  | A_33_P3369495 | 4.554 | CLDN10 | claudin 10 [Source:HGNC Symbol;Acc:2033] [ENST00000376855] |
|  |  | A_33_P3406873 | 4.192 | NFASC | Homo sapiens neurofascin (NFASC), transcript variant 5, mRNA [NM_001005389] |
|  |  | A_33_P3651948 | 3.305 | NEO1 | Homo sapiens neogenin 1 (NEO1), transcript variant 1, mRNA [NM_002499] |
| Glycine, serine and threonine metabolism | 0.003 | A_23_P129064 | -2.969 | GATM | Homo sapiens glycine amidinotransferase (L-arginine:glycine amidinotransferase) (GATM), nuclear gene encoding mitochondrial protein, mRNA [NM_001482] |
|  |  | A_23_P164258 | 2.836 | PIPOX | Homo sapiens pipecolic acid oxidase (PIPOX), mRNA [NM_016518] |
|  |  | A_23_P2645 | 2.052 | SDS | Homo sapiens serine dehydratase (SDS), mRNA [NM_006843] |
|  |  | A_23_P40657 | 2.367 | GCAT | Homo sapiens glycine C-acetyltransferase (GCAT), nuclear gene encoding mitochondrial protein, transcript variant 2, mRNA [NM_014291] |
|  |  | A_23_P4133 | 2.416 | AOC2 | Homo sapiens amine oxidase, copper containing 2 (retina-specific) (AOC2), transcript variant 1, mRNA [NM_001158] |
|  |  | A_23_P85015 | 2.858 | MAOB | Homo sapiens monoamine oxidase B (MAOB), nuclear gene encoding mitochondrial protein, mRNA [NM_000898] |
|  |  | A_23_P85783 | -2.611 | PHGDH | Homo sapiens phosphoglycerate dehydrogenase (PHGDH), mRNA [NM_006623] |
|  |  | A_32_P385587 | 3.930 | ALAS2 | Homo sapiens aminolevulinate, delta-, synthase 2 (ALAS2), nuclear gene encoding mitochondrial protein, transcript variant 1, mRNA [NM_000032] |
|  |  | A_33_P3242543 | 5.810 | MAOA | Homo sapiens monoamine oxidase A (MAOA), nuclear gene encoding mitochondrial protein, mRNA [NM_000240] |
|  |  | A_33_P3250278 | 2.054 | AGXT | Homo sapiens alanine-glyoxylate aminotransferase (AGXT), mRNA [NM_000030] |
|  |  | A_33_P3257861 | -2.486 | SARDH | Homo sapiens sarcosine dehydrogenase (SARDH), nuclear gene encoding mitochondrial protein, transcript variant 2, mRNA [NM_001134707] |
| ECM-receptor interaction | 0.007 | A_23_P109034 | 2.056 | SDC4 | Homo sapiens syndecan 4 (SDC4), mRNA [NM_002999] |
|  |  | A_23_P23728 | 3.645 | SV2A | Homo sapiens synaptic vesicle glycoprotein 2A (SV2A), mRNA [NM_014849] |
|  |  | A_23_P252193 | 2.086 | ITGA9 | Homo sapiens integrin, alpha 9 (ITGA9), mRNA [NM_002207] |
|  |  | A_23_P26976 | 2.108 | CHAD | Homo sapiens chondroadherin (CHAD), mRNA [NM_001267] |
|  |  | A_23_P310956 | -3.527 | COL6A2 | Homo sapiens collagen, type VI, alpha 2 (COL6A2), transcript variant 2C2a', mRNA [NM_058175] |
|  |  | A_23_P343411 | 4.488 | AGRN | Homo sapiens agrin (AGRN), mRNA [NM_198576] |
|  |  | A_23_P55749 | -2.818 | COL5A3 | Homo sapiens collagen, type V, alpha 3 (COL5A3), mRNA [NM_015719] |
|  |  | A_23_P82979 | 2.590 | LAMC3 | Homo sapiens laminin, gamma 3 (LAMC3), mRNA [NM_006059] |
|  |  | A_23_P89780 | 3.355 | LAMA3 | Homo sapiens laminin, alpha 3 (LAMA3), transcript variant 1, mRNA [NM_198129] |
|  |  | A_24_P944964 | -2.211 | GP5 | Homo sapiens glycoprotein V (platelet) (GP5), mRNA [NM_004488] |
|  |  | A_33_P3216448 | 6.215 | COL11A2 | Homo sapiens collagen, type XI, alpha 2 (COL11A2), transcript variant 4, mRNA [NM_001163771] |
|  |  | A_33_P3227400 | -2.207 | COL4A4 | Homo sapiens collagen, type IV, alpha 4 (COL4A4), mRNA [NM_000092] |
|  |  | A_33_P3231447 | -2.528 | ITGA6 | Homo sapiens integrin, alpha 6 (ITGA6), transcript variant 2, mRNA [NM_000210] |
|  |  | A_33_P3262969 | 7.530 | COL4A6 | collagen, type IV, alpha 6 [Source:HGNC Symbol;Acc:2208] [ENST00000461897] |
|  |  | A_33_P3314301 | 3.018 | SV2C | Homo sapiens synaptic vesicle glycoprotein 2C (SV2C), mRNA [NM_014979] |
|  |  | A_33_P3370930 | 2.973 | LAMB1 | laminin, beta 1 [Source:HGNC Symbol;Acc:6486] [ENST00000393559] |
|  |  | A_33_P3380618 | 2.204 | HSPG2 | Homo sapiens heparan sulfate proteoglycan 2 (HSPG2), mRNA [NM_005529] |
|  |  | A_33_P3381338 | 2.283 | TNXB | Homo sapiens tenascin XB (TNXB), transcript variant XB, mRNA [NM_019105] |
|  |  | A_33_P3415820 | 3.170 | THBS1 | Homo sapiens thrombospondin 1 (THBS1), mRNA [NM_003246] |
|  |  | A_33_P3629678 | 4.382 | COL5A1 | Homo sapiens collagen, type V, alpha 1 (COL5A1), mRNA [NM_000093] |
| Phenylalanine metabolism | 0.015 | A_23_P139527 | 2.107 | HPD | Homo sapiens 4-hydroxyphenylpyruvate dioxygenase (HPD), transcript variant 1, mRNA [NM_002150] |
|  |  | A_23_P4133 | 2.416 | AOC2 | Homo sapiens amine oxidase, copper containing 2 (retina-specific) (AOC2), transcript variant 1, mRNA [NM_001158] |
|  |  | A_23_P85015 | 2.858 | MAOB | Homo sapiens monoamine oxidase B (MAOB), nuclear gene encoding mitochondrial protein, mRNA [NM_000898] |
|  |  | A_23_P983 | 3.211 | PRDX6 | Homo sapiens peroxiredoxin 6 (PRDX6), mRNA [NM_004905] |
|  |  | A_24_P330633 | 8.676 | TAT | Homo sapiens tyrosine aminotransferase (TAT), nuclear gene encoding mitochondrial protein, mRNA [NM_000353] |
|  |  | A_33_P3238433 | 2.068 | ALDH3A1 | Homo sapiens aldehyde dehydrogenase 3 family, member A1 (ALDH3A1), transcript variant 1, mRNA [NM_001135168] |
|  |  | A_33_P3242543 | 5.810 | MAOA | Homo sapiens monoamine oxidase A (MAOA), nuclear gene encoding mitochondrial protein, mRNA [NM_000240] |
|  |  | A_33_P3367447 | 2.652 | ALDH3B1 | Homo sapiens aldehyde dehydrogenase 3 family, member B1 (ALDH3B1), transcript variant 3, mRNA [NM_001161473] |
| Tyrosine metabolism | 0.017 | A_23_P100642 | -2.572 | PNMT | Homo sapiens phenylethanolamine N-methyltransferase (PNMT), mRNA [NM_002686] |
|  |  | A_23_P139527 | 2.107 | HPD | Homo sapiens 4-hydroxyphenylpyruvate dioxygenase (HPD), transcript variant 1, mRNA [NM_002150] |
|  |  | A_23_P250164 | -2.254 | HGD | Homo sapiens homogentisate 1,2-dioxygenase (HGD), mRNA [NM_000187] |
|  |  | A_23_P4133 | 2.416 | AOC2 | Homo sapiens amine oxidase, copper containing 2 (retina-specific) (AOC2), transcript variant 1, mRNA [NM_001158] |
|  |  | A_23_P76622 | 2.993 | DCT | Homo sapiens dopachrome tautomerase (dopachrome delta-isomerase, tyrosine-related protein 2) (DCT), transcript variant 1, mRNA [NM_001922] |
|  |  | A_23_P85015 | 2.858 | MAOB | Homo sapiens monoamine oxidase B (MAOB), nuclear gene encoding mitochondrial protein, mRNA [NM_000898] |
|  |  | A_24_P268729 | -2.105 | HEMK1 | Homo sapiens HemK methyltransferase family member 1 (HEMK1), mRNA [NM_016173] |
|  |  | A_24_P330633 | 8.676 | TAT | Homo sapiens tyrosine aminotransferase (TAT), nuclear gene encoding mitochondrial protein, mRNA [NM_000353] |
|  |  | A_33_P3238433 | 2.068 | ALDH3A1 | Homo sapiens aldehyde dehydrogenase 3 family, member A1 (ALDH3A1), transcript variant 1, mRNA [NM_001135168] |
|  |  | A_33_P3242543 | 5.810 | MAOA | Homo sapiens monoamine oxidase A (MAOA), nuclear gene encoding mitochondrial protein, mRNA [NM_000240] |
|  |  | A_33_P3252414 | 2.230 | TH | Homo sapiens tyrosine hydroxylase (TH), transcript variant 1, mRNA [NM_199292] |
|  |  | A_33_P3367447 | 2.652 | ALDH3B1 | Homo sapiens aldehyde dehydrogenase 3 family, member B1 (ALDH3B1), transcript variant 3, mRNA [NM_001161473] |
| Purine metabolism | 0.039 | A_23_P117580 | 2.305 | ENTPD5 | Homo sapiens ectonucleoside triphosphate diphosphohydrolase 5 (ENTPD5), mRNA [NM_001249] |
|  |  | A_23_P201022 | 2.582 | PKLR | Homo sapiens pyruvate kinase, liver and RBC (PKLR), nuclear gene encoding mitochondrial protein, transcript variant 1, mRNA [NM_000298] |
|  |  | A_23_P201097 | 2.952 | GUK1 | Homo sapiens guanylate kinase 1 (GUK1), transcript variant 2, mRNA [NM_000858] |
|  |  | A_23_P215669 | 2.895 | POLR2J2 | Homo sapiens polymerase (RNA) II (DNA directed) polypeptide J2 (POLR2J2), mRNA [NM_032959] |
|  |  | A_23_P29096 | -2.810 | PDE9A | Homo sapiens phosphodiesterase 9A (PDE9A), transcript variant 1, mRNA [NM_002606] |
|  |  | A_23_P36928 | 2.305 | POLR1D | Homo sapiens polymerase (RNA) I polypeptide D, 16kDa (POLR1D), transcript variant 1, mRNA [NM_015972] |
|  |  | A_23_P401106 | 2.047 | PDE2A | Homo sapiens phosphodiesterase 2A, cGMP-stimulated (PDE2A), transcript variant 1, mRNA [NM_002599] |
|  |  | A_23_P402610 | -2.885 | PFAS | Homo sapiens phosphoribosylformylglycinamidine synthase (PFAS), mRNA [NM_012393] |
|  |  | A_23_P80940 | -2.129 | PPAT | Homo sapiens phosphoribosyl pyrophosphate amidotransferase (PPAT), mRNA [NM_002703] |
|  |  | A_23_P9458 | -2.043 | POLR1E | Homo sapiens polymerase (RNA) I polypeptide E, 53kDa (POLR1E), mRNA [NM_022490] |
|  |  | A_24_P113960 | 3.258 | NT5C1A | Homo sapiens 5'-nucleotidase, cytosolic IA (NT5C1A), mRNA [NM_032526] |
|  |  | A_24_P208436 | 2.502 | PDE1A | Homo sapiens phosphodiesterase 1A, calmodulin-dependent (PDE1A), transcript variant 2, mRNA [NM_001003683] |
|  |  | A_24_P277657 | 4.205 | GMPR | Homo sapiens guanosine monophosphate reductase (GMPR), mRNA [NM_006877] |
|  |  | A_24_P278192 | 5.693 | AK1 | Homo sapiens adenylate kinase 1 (AK1), mRNA [NM_000476] |
|  |  | A_24_P316430 | -2.036 | NT5E | Homo sapiens 5'-nucleotidase, ecto (CD73) (NT5E), transcript variant 1, mRNA [NM_002526] |
|  |  | A_24_P342632 | -2.573 | AK5 | Homo sapiens adenylate kinase 5 (AK5), transcript variant 1, mRNA [NM_174858] |
|  |  | A_32_P834166 | 4.342 | LOC100128843 | Homo sapiens DNA polymerase epsilon catalytic subunit isoform a (POLE1) mRNA, partial cds. [AF128541] |
|  |  | A_33_P3214298 | -2.062 | IMPDH2 | Homo sapiens IMP (inosine 5'-monophosphate) dehydrogenase 2 (IMPDH2), mRNA [NM_000884] |
|  |  | A_33_P3216601 | -2.234 | FHIT | Homo sapiens fragile histidine triad gene (FHIT), transcript variant 1, mRNA [NM_002012] |
|  |  | A_33_P3216938 | 3.726 | AMPD3 | adenosine monophosphate deaminase 3 [Source:HGNC Symbol;Acc:470] [ENST00000527261] |
|  |  | A_33_P3242863 | 2.177 | NT5M | Homo sapiens 5',3'-nucleotidase, mitochondrial (NT5M), nuclear gene encoding mitochondrial protein, mRNA [NM_020201] |
|  |  | A_33_P3262580 | 2.350 | ENTPD1 | ectonucleoside triphosphate diphosphohydrolase 1 [Source:HGNC Symbol;Acc:3363] [ENST00000371206] |
|  |  | A_33_P3318963 | 3.847 | POLR2F | polymerase (RNA) II (DNA directed) polypeptide F [Source:HGNC Symbol;Acc:9193] [ENST00000405557] |
|  |  | A_33_P3320217 | 3.002 | ADCY2 | Homo sapiens adenylate cyclase 2 (brain) (ADCY2), mRNA [NM_020546] |
|  |  | A_33_P3337161 | 2.605 | ADCY4 | Homo sapiens adenylate cyclase 4 (ADCY4), transcript variant 3, mRNA [NM_001198568] |
|  |  | A_33_P3395876 | 2.302 | PDE4A | Homo sapiens phosphodiesterase 4A, cAMP-specific (PDE4A), transcript variant 1, mRNA [NM_001111307] |
|  |  | A_33_P3396459 | 4.168 | POLR2H | polymerase (RNA) II (DNA directed) polypeptide H [Source:HGNC Symbol;Acc:9195] [ENST00000412877] |
|  |  | A_33_P3759611 | 2.015 | PDE4C | Homo sapiens phosphodiesterase 4C, cAMP-specific (PDE4C), transcript variant 1, mRNA [NM_000923] |
| TGF-beta signaling pathway | 0.039 | A_23_P160336 | -2.228 | LEFTY1 | Homo sapiens left-right determination factor 1 (LEFTY1), mRNA [NM_020997] |
|  |  | A_23_P215956 | -2.022 | MYC | Homo sapiens v-myc myelocytomatosis viral oncogene homolog (avian) (MYC), mRNA [NM_002467] |
|  |  | A_23_P259955 | 2.556 | GDF5 | Homo sapiens growth differentiation factor 5 (GDF5), mRNA [NM_000557] |
|  |  | A_23_P2814 | 2.979 | SMAD9 | Homo sapiens SMAD family member 9 (SMAD9), transcript variant b, mRNA [NM_005905] |
|  |  | A_23_P397455 | -2.180 | ACVR1C | Homo sapiens activin A receptor, type IC (ACVR1C), transcript variant 1, mRNA [NM_145259] |
|  |  | A_23_P502047 | 2.578 | CHRD | Homo sapiens chordin (CHRD), mRNA [NM_003741] |
|  |  | A_24_P141707 | 3.696 | INHBE | Homo sapiens inhibin, beta E (INHBE), mRNA [NM_031479] |
|  |  | A_24_P155502 | 3.345 | INHBC | Homo sapiens inhibin, beta C (INHBC), mRNA [NM_005538] |
|  |  | A_24_P945113 | 2.034 | ACVRL1 | Homo sapiens activin A receptor type II-like 1 (ACVRL1), transcript variant 1, mRNA [NM_000020] |
|  |  | A_32_P143048 | -2.389 | ZFYVE9 | Homo sapiens zinc finger, FYVE domain containing 9 (ZFYVE9), transcript variant 3, mRNA [NM_004799] |
|  |  | A_32_P199301 | 3.392 | TFDP1 | Homo sapiens transcription factor Dp-1 (TFDP1), transcript variant 1, mRNA [NM_007111] |
|  |  | A_33_P3220445 | 2.391 | SMAD5 | Homo sapiens SMAD family member 5 (SMAD5), transcript variant 2, mRNA [NM_001001419] |
|  |  | A_33_P3246318 | 2.746 | RBX1 | Homo sapiens ring-box 1, E3 ubiquitin protein ligase (RBX1), mRNA [NM_014248] |
|  |  | A_33_P3304655 | 2.846 | LTBP1 | Homo sapiens latent transforming growth factor beta binding protein 1 (LTBP1), transcript variant 1, mRNA [NM_206943] |
|  |  | A_33_P3364661 | 2.064 | RHOA | ras homolog gene family, member A [Source:HGNC Symbol;Acc:667] [ENST00000265538] |
|  |  | A_33_P3391796 | -4.521 | NOG | Homo sapiens noggin (NOG), mRNA [NM_005450] |
|  |  | A_33_P3406240 | 2.778 | GDF7 | Homo sapiens growth differentiation factor 7 (GDF7), mRNA [NM_182828] |
|  |  | A_33_P3415820 | 3.170 | THBS1 | Homo sapiens thrombospondin 1 (THBS1), mRNA [NM_003246] |
| Arachidonic acid metabolism | 0.041 | A_23_P103465 | 2.209 | PLA2G5 | Homo sapiens phospholipase A2, group V (PLA2G5), mRNA [NM_000929] |
|  |  | A_23_P103486 | -2.407 | CYP2J2 | Homo sapiens cytochrome P450, family 2, subfamily J, polypeptide 2 (CYP2J2), mRNA [NM_000775] |
|  |  | A_23_P3038 | 3.025 | GPX2 | Homo sapiens glutathione peroxidase 2 (gastrointestinal) (GPX2), mRNA [NM_002083] |
|  |  | A_23_P321949 | 3.150 | PLA2G2A | Homo sapiens phospholipase A2, group IIA (platelets, synovial fluid) (PLA2G2A), transcript variant 1, mRNA [NM_000300] |
|  |  | A_23_P8834 | -2.920 | EPHX2 | Homo sapiens epoxide hydrolase 2, cytoplasmic (EPHX2), mRNA [NM_001979] |
|  |  | A_24_P191013 | 4.419 | CYP4A11 | Homo sapiens cytochrome P450, family 4, subfamily A, polypeptide 11 (CYP4A11), mRNA [NM_000778] |
|  |  | A_24_P339514 | 4.763 | CYP2B6 | Homo sapiens cytochrome P450, family 2, subfamily B, polypeptide 6 (CYP2B6), mRNA [NM_000767] |
|  |  | A_33_P3220149 | 2.729 | MAML1 | mastermind-like 1 (Drosophila) [Source:HGNC Symbol;Acc:13632] [ENST00000503050] |
|  |  | A_33_P3239849 | 3.534 | GPX1 | Homo sapiens glutathione peroxidase 1 (GPX1), transcript variant 2, mRNA [NM_201397] |
|  |  | A_33_P3255304 | 2.101 | GGT5 | Homo sapiens gamma-glutamyltransferase 5 (GGT5), transcript variant 1, mRNA [NM_001099781] |
|  |  | A_33_P3275600 | 3.672 | PLA2G4E | Homo sapiens phospholipase A2, group IVE (PLA2G4E), mRNA [NM_001206670] |
| ABC transporters | 0.042 | A_23_P140876 | -2.417 | ABCA3 | Homo sapiens ATP-binding cassette, sub-family A (ABC1), member 3 (ABCA3), mRNA [NM_001089] |
|  |  | A_23_P24774 | 2.016 | ABCC8 | Homo sapiens ATP-binding cassette, sub-family C (CFTR/MRP), member 8 (ABCC8), mRNA [NM_000352] |
|  |  | A_23_P401361 | -2.560 | PITPNM2 | Homo sapiens phosphatidylinositol transfer protein, membrane-associated 2 (PITPNM2), mRNA [NM_020845] |
|  |  | A_24_P16913 | 2.780 | ABCC4 | Homo sapiens ATP-binding cassette, sub-family C (CFTR/MRP), member 4 (ABCC4), transcript variant 1, mRNA [NM_005845] |
|  |  | A_33_P3236686 | -2.055 |  | Homo sapiens FP15331 mRNA, complete cds. [AF461897] |
|  |  | A_33_P3251672 | 2.924 | ABCA2 | Homo sapiens ATP-binding cassette, sub-family A (ABC1), member 2 (ABCA2), transcript variant 1, mRNA [NM_001606] |
|  |  | A_33_P3298057 | 2.144 | ABCC5 | Homo sapiens ATP-binding cassette, sub-family C (CFTR/MRP), member 5 (ABCC5), transcript variant 2, mRNA [NM_001023587] |
|  |  | A_33_P3332414 | -2.022 | ABCB1 | Homo sapiens ATP-binding cassette, sub-family B (MDR/TAP), member 1 (ABCB1), mRNA [NM_000927] |
|  |  | A_33_P3336780 | -2.099 | ABCB8 | Homo sapiens ATP-binding cassette, sub-family B (MDR/TAP), member 8 (ABCB8), nuclear gene encoding mitochondrial protein, mRNA [NM_007188] |
|  |  | A_33_P3361067 | 2.707 | ABCG2 | Homo sapiens ATP-binding cassette, sub-family G (WHITE), member 2 (ABCG2), mRNA [NM_004827] |
|  |  | A_33_P3372563 | 4.358 | ABCC10 | Homo sapiens ATP-binding cassette, sub-family C (CFTR/MRP), member 10 (ABCC10), transcript variant MRP7A, mRNA [NM_033450] |
|  |  | A_33_P3422897 | 2.440 | ABCA1 | Homo sapiens ATP-binding cassette, sub-family A (ABC1), member 1 (ABCA1), mRNA [NM_005502] |
| Cardiac muscle contraction | 0.057 | A_23_P110811 | 2.266 | COX7C | Homo sapiens cytochrome c oxidase subunit VIIc (COX7C), nuclear gene encoding mitochondrial protein, mRNA [NM_001867] |
|  |  | A_23_P159650 | 2.856 | COX7B | Homo sapiens cytochrome c oxidase subunit VIIb (COX7B), nuclear gene encoding mitochondrial protein, mRNA [NM_001866] |
|  |  | A_23_P31671 | 2.335 | UQCRB | Homo sapiens ubiquinol-cytochrome c reductase binding protein (UQCRB), nuclear gene encoding mitochondrial protein, transcript variant 1, mRNA [NM_006294] |
|  |  | A_23_P414328 | 3.074 | CACNG5 | Homo sapiens calcium channel, voltage-dependent, gamma subunit 5 (CACNG5), mRNA [NM_145811] |
|  |  | A_23_P420218 | 2.845 | CACNG8 | Homo sapiens calcium channel, voltage-dependent, gamma subunit 8 (CACNG8), mRNA [NM_031895] |
|  |  | A_23_P501933 | -2.095 | CACNG6 | Homo sapiens calcium channel, voltage-dependent, gamma subunit 6 (CACNG6), transcript variant 1, mRNA [NM_145814] |
|  |  | A_23_P56314 | 3.355 | UQCR11 | Homo sapiens ubiquinol-cytochrome c reductase, complex III subunit XI (UQCR11), nuclear gene encoding mitochondrial protein, mRNA [NM_006830] |
|  |  | A_23_P78571 | 2.879 | COX6B2 | Homo sapiens cytochrome c oxidase subunit VIb polypeptide 2 (testis) (COX6B2), mRNA [NM_144613] |
|  |  | A_23_P85765 | 2.001 | CACNA1S | Homo sapiens calcium channel, voltage-dependent, L type, alpha 1S subunit (CACNA1S), mRNA [NM_000069] |
|  |  | A_24_P31275 | 3.105 | ATP1B2 | Homo sapiens ATPase, Na+/K+ transporting, beta 2 polypeptide (ATP1B2), mRNA [NM_001678] |
|  |  | A_24_P70303 | 2.029 | CACNG4 | Homo sapiens calcium channel, voltage-dependent, gamma subunit 4 (CACNG4), mRNA [NM_014405] |
|  |  | A_33_P3221989 | -2.450 | CACNB4 | Homo sapiens calcium channel, voltage-dependent, beta 4 subunit (CACNB4), transcript variant 1, mRNA [NM_001005747] |
|  |  | A_33_P3262156 | 3.035 | SLC8A1 | Homo sapiens solute carrier family 8 (sodium/calcium exchanger), member 1 (SLC8A1), transcript variant A, mRNA [NM_021097] |
|  |  | A_33_P3270599 | -2.420 | TPM2 | Homo sapiens tropomyosin 2 (beta) (TPM2), transcript variant 2, mRNA [NM_213674] |
|  |  | A_33_P3283669 | 2.727 | ATP1A3 | Homo sapiens ATPase, Na+/K+ transporting, alpha 3 polypeptide (ATP1A3), mRNA [NM_152296] |
|  |  | A_33_P3335966 | 3.010 | TPM1 | Homo sapiens tropomyosin 1 (alpha) (TPM1), transcript variant 1, mRNA [NM_001018005] |
| Dilated cardiomyopathy | 0.062 | A_23_P252193 | 2.086 | ITGA9 | Homo sapiens integrin, alpha 9 (ITGA9), mRNA [NM_002207] |
|  |  | A_23_P414328 | 3.074 | CACNG5 | Homo sapiens calcium channel, voltage-dependent, gamma subunit 5 (CACNG5), mRNA [NM_145811] |
|  |  | A_23_P420218 | 2.845 | CACNG8 | Homo sapiens calcium channel, voltage-dependent, gamma subunit 8 (CACNG8), mRNA [NM_031895] |
|  |  | A_23_P501933 | -2.095 | CACNG6 | Homo sapiens calcium channel, voltage-dependent, gamma subunit 6 (CACNG6), transcript variant 1, mRNA [NM_145814] |
|  |  | A_23_P85269 | -2.425 | TTN | Homo sapiens titin (TTN), transcript variant N2-A, mRNA [NM_133378] |
|  |  | A_23_P85765 | 2.001 | CACNA1S | Homo sapiens calcium channel, voltage-dependent, L type, alpha 1S subunit (CACNA1S), mRNA [NM_000069] |
|  |  | A_24_P168574 | 2.125 | GNAS | GNAS complex locus [Source:HGNC Symbol;Acc:4392] [ENST00000481768] |
|  |  | A_24_P70303 | 2.029 | CACNG4 | Homo sapiens calcium channel, voltage-dependent, gamma subunit 4 (CACNG4), mRNA [NM_014405] |
|  |  | A_33_P3221989 | -2.450 | CACNB4 | Homo sapiens calcium channel, voltage-dependent, beta 4 subunit (CACNB4), transcript variant 1, mRNA [NM_001005747] |
|  |  | A_33_P3231447 | -2.528 | ITGA6 | Homo sapiens integrin, alpha 6 (ITGA6), transcript variant 2, mRNA [NM_000210] |
|  |  | A_33_P3262156 | 3.035 | SLC8A1 | Homo sapiens solute carrier family 8 (sodium/calcium exchanger), member 1 (SLC8A1), transcript variant A, mRNA [NM_021097] |
|  |  | A_33_P3270599 | -2.420 | TPM2 | Homo sapiens tropomyosin 2 (beta) (TPM2), transcript variant 2, mRNA [NM_213674] |
|  |  | A_33_P3275801 | 2.913 | DES | Homo sapiens desmin (DES), mRNA [NM_001927] |
|  |  | A_33_P3284763 | 3.029 | DMD | Homo sapiens dystrophin (DMD), transcript variant Dp140b, mRNA [NM_004021] |
|  |  | A_33_P3311285 | 4.553 | LMNA | Homo sapiens lamin A/C (LMNA), transcript variant 1, mRNA [NM_170707] |
|  |  | A_33_P3320217 | 3.002 | ADCY2 | Homo sapiens adenylate cyclase 2 (brain) (ADCY2), mRNA [NM_020546] |
|  |  | A_33_P3335966 | 3.010 | TPM1 | Homo sapiens tropomyosin 1 (alpha) (TPM1), transcript variant 1, mRNA [NM_001018005] |
|  |  | A_33_P3337161 | 2.605 | ADCY4 | Homo sapiens adenylate cyclase 4 (ADCY4), transcript variant 3, mRNA [NM_001198568] |
| Primary immunodeficiency | 0.063 | A_23_P107735 | 2.947 | CD79A | Homo sapiens CD79a molecule, immunoglobulin-associated alpha (CD79A), transcript variant 1, mRNA [NM_001783] |
|  |  | A_23_P159335 | -2.899 | CD8B | Homo sapiens CD8b molecule (CD8B), transcript variant 5, mRNA [NM_004931] |
|  |  | A_23_P329112 | 2.016 | JAK3 | Homo sapiens Janus kinase 3 (JAK3), mRNA [NM_000215] |
|  |  | A_23_P404494 | -2.201 | IL7R | Homo sapiens interleukin 7 receptor (IL7R), mRNA [NM_002185] |
|  |  | A_23_P84705 | -2.090 | TNFRSF13B | Homo sapiens tumor necrosis factor receptor superfamily, member 13B (TNFRSF13B), mRNA [NM_012452] |
|  |  | A_24_P169234 | -2.149 | ZAP70 | Homo sapiens zeta-chain (TCR) associated protein kinase 70kDa (ZAP70), transcript variant 1, mRNA [NM_001079] |
|  |  | A_24_P295999 | 2.708 | CD4 | Homo sapiens CD4 molecule (CD4), transcript variant 1, mRNA [NM_000616] |
|  |  | A_32_P163247 | -2.656 | CD8A | Homo sapiens CD8a molecule (CD8A), transcript variant 1, mRNA [NM_001768] |
|  |  | A_33_P3250680 | -2.369 | CD40LG | Homo sapiens CD40 ligand (CD40LG), mRNA [NM_000074] |
| Axon guidance | 0.079 | A_23_P108501 | -2.186 | EPHA4 | Homo sapiens EPH receptor A4 (EPHA4), mRNA [NM_004438] |
|  |  | A_23_P113005 | 2.407 | EFNA1 | Homo sapiens ephrin-A1 (EFNA1), transcript variant 1, mRNA [NM_004428] |
|  |  | A_23_P114057 | -2.049 | SEMA4C | Homo sapiens sema domain, immunoglobulin domain (Ig), transmembrane domain (TM) and short cytoplasmic domain, (semaphorin) 4C (SEMA4C), mRNA [NM_017789] |
|  |  | A_23_P119535 | 2.976 | EFNA2 | Homo sapiens ephrin-A2 (EFNA2), mRNA [NM_001405] |
|  |  | A_23_P157333 | -2.447 | EPHA1 | Homo sapiens EPH receptor A1 (EPHA1), mRNA [NM_005232] |
|  |  | A_23_P162449 | 2.794 | SRGAP1 | Homo sapiens SLIT-ROBO Rho GTPase activating protein 1 (SRGAP1), mRNA [NM_020762] |
|  |  | A_23_P202520 | -2.772 | ABLIM1 | Homo sapiens actin binding LIM protein 1 (ABLIM1), transcript variant 3, mRNA [NM_001003408] |
|  |  | A_23_P57667 | -2.403 | PLXNA1 | Homo sapiens plexin A1 (PLXNA1), mRNA [NM_032242] |
|  |  | A_23_P69617 | 4.221 | UNC5C | Homo sapiens unc-5 homolog C (C. elegans) (UNC5C), mRNA [NM_003728] |
|  |  | A_23_P87082 | 3.123 | ROBO3 | Homo sapiens roundabout, axon guidance receptor, homolog 3 (Drosophila) (ROBO3), mRNA [NM_022370] |
|  |  | A_23_P95165 | 2.142 | SEMA4B | Homo sapiens sema domain, immunoglobulin domain (Ig), transmembrane domain (TM) and short cytoplasmic domain, (semaphorin) 4B (SEMA4B), transcript variant 1, mRNA [NM_020210] |
|  |  | A_24_P114032 | 2.136 | EFNA3 | Homo sapiens ephrin-A3 (EFNA3), mRNA [NM_004952] |
|  |  | A_24_P313822 | 2.292 | PAK4 | Homo sapiens p21 protein (Cdc42/Rac)-activated kinase 4 (PAK4), transcript variant 1, mRNA [NM_005884] |
|  |  | A_32_P195065 | -2.249 | SEMA4F | Homo sapiens sema domain, immunoglobulin domain (Ig), transmembrane domain (TM) and short cytoplasmic domain, (semaphorin) 4F (SEMA4F), mRNA [NM_004263] |
|  |  | A_33_P3220160 | 2.115 | RAC2 | Homo sapiens cDNA FLJ39605 fis, clone SKNSH2005981, weakly similar to RAS-RELATED C3 BOTULINUM TOXIN SUBSTRATE 2. [AK096924] |
|  |  | A_33_P3288609 | 2.646 | PAK1 | p21 protein (Cdc42/Rac)-activated kinase 1 [Source:HGNC Symbol;Acc:8590] [ENST00000526968] |
|  |  | A_33_P3364661 | 2.064 | RHOA | ras homolog gene family, member A [Source:HGNC Symbol;Acc:667] [ENST00000265538] |
|  |  | A_33_P3368540 | 2.056 |  | SLIT-ROBO Rho GTPase activating protein 3 [Source:HGNC Symbol;Acc:19744] [ENST00000490889] |
|  |  | A_33_P3370787 | 2.658 | EPHB2 | Homo sapiens EPH receptor B2 (EPHB2), transcript variant 2, mRNA [NM_004442] |
|  |  | A_33_P3379001 | 2.902 | UNC5A | Homo sapiens unc-5 homolog A (C. elegans) (UNC5A), mRNA [NM_133369] |
|  |  | A_33_P3413048 | -2.688 | PLXNA3 | Homo sapiens plexin A3 (PLXNA3), mRNA [NM_017514] |
|  |  | A_33_P3416707 | 2.090 | PTK2 | PTK2 protein tyrosine kinase 2 [Source:HGNC Symbol;Acc:9611] [ENST00000342207] |
|  |  | A_33_P3712341 | 3.284 | CXCL12 | Homo sapiens chemokine (C-X-C motif) ligand 12 (CXCL12), transcript variant 3, mRNA [NM_001033886] |
| Adherens junction | 0.095 | A_23_P301304 | -2.128 | FGFR1 | Homo sapiens fibroblast growth factor receptor 1 (FGFR1), transcript variant 1, mRNA [NM_023110] |
|  |  | A_23_P308603 | 2.223 | SRC | Homo sapiens v-src sarcoma (Schmidt-Ruppin A-2) viral oncogene homolog (avian) (SRC), transcript variant 1, mRNA [NM_005417] |
|  |  | A_23_P315836 | -2.193 | BAIAP2 | Homo sapiens BAI1-associated protein 2 (BAIAP2), transcript variant 2, mRNA [NM_017451] |
|  |  | A_23_P397455 | -2.180 | ACVR1C | Homo sapiens activin A receptor, type IC (ACVR1C), transcript variant 1, mRNA [NM_145259] |
|  |  | A_23_P7582 | -2.547 | TCF7 | Homo sapiens transcription factor 7 (T-cell specific, HMG-box) (TCF7), transcript variant 1, mRNA [NM_003202] |
|  |  | A_23_P76034 | 2.721 | PVRL1 | Homo sapiens poliovirus receptor-related 1 (herpesvirus entry mediator C) (PVRL1), transcript variant 3, mRNA [NM_203286] |
|  |  | A_24_P20630 | -2.738 | LEF1 | Homo sapiens lymphoid enhancer-binding factor 1 (LEF1), transcript variant 1, mRNA [NM_016269] |
|  |  | A_24_P317907 | 4.794 | SORBS1 | Homo sapiens sorbin and SH3 domain containing 1 (SORBS1), transcript variant 3, mRNA [NM_001034954] |
|  |  | A_24_P385313 | 2.583 | PTPRF | Homo sapiens protein tyrosine phosphatase, receptor type, F (PTPRF), transcript variant 1, mRNA [NM_002840] |
|  |  | A_33_P3220160 | 2.115 | RAC2 | Homo sapiens cDNA FLJ39605 fis, clone SKNSH2005981, weakly similar to RAS-RELATED C3 BOTULINUM TOXIN SUBSTRATE 2. [AK096924] |
|  |  | A_33_P3243812 | 2.459 | WASF1 | Homo sapiens WAS protein family, member 1 (WASF1), transcript variant 1, mRNA [NM_003931] |
|  |  | A_33_P3280801 | -2.218 | LMO7 | Homo sapiens LIM domain 7 (LMO7), transcript variant 1, mRNA [NM_005358] |
|  |  | A_33_P3303372 | -2.007 | PARD3 | Homo sapiens par-3 partitioning defective 3 homolog (C. elegans) (PARD3), transcript variant 9, mRNA [NM_001184792] |
|  |  | A_33_P3319155 | 2.273 | ACP1 | Homo sapiens acid phosphatase 1, soluble (ACP1), transcript variant 4, mRNA [NM_001040649] |
|  |  | A_33_P3364661 | 2.064 | RHOA | ras homolog gene family, member A [Source:HGNC Symbol;Acc:667] [ENST00000265538] |
